# Intricate Crosstalk Between Lipopolysaccharide, Phospholipid and Fatty Acid Metabolism in *Escherichia coli* Modulates Proteolysis of LpxC

**DOI:** 10.3389/fmicb.2018.03285

**Published:** 2019-01-14

**Authors:** Nikolas Thomanek, Jan Arends, Claudia Lindemann, Katalin Barkovits, Helmut E. Meyer, Katrin Marcus, Franz Narberhaus

**Affiliations:** ^1^Medical Proteome Center, Ruhr University Bochum, Bochum, Germany; ^2^Microbial Biology, Ruhr University Bochum, Bochum, Germany; ^3^Biomedical Research, Leibniz-Institut für Analytische Wissenschaften – ISAS – e. V., Dortmund, Germany

**Keywords:** lipopolysaccharide, phospholipid, proteolysis, super-SILAC, quantitative proteomics, ppGpp, *Escherichia coli*

## Abstract

Lipopolysaccharides (LPS) in the outer membrane of Gram-negative bacteria provide the first line of defense against antibiotics and other harmful compounds. LPS biosynthesis critically depends on LpxC catalyzing the first committed enzyme in this process. In *Escherichia coli*, the cellular concentration of LpxC is adjusted in a growth rate-dependent manner by the FtsH protease making sure that LPS biosynthesis is coordinated with the cellular demand. As a result, LpxC is stable in fast-growing cells and prone to degradation in slow-growing cells. One of the factors involved in this process is the alarmone guanosine tetraphosphate (ppGpp) but previous studies suggested the involvement of yet unknown factors in LpxC degradation. We established a quantitative proteomics approach aiming at the identification of proteins that are associated with LpxC and/or FtsH at high or low growth rates. The identification of known LpxC and FtsH interactors validated our approach. A number of proteins involved in fatty acid biosynthesis and degradation, including the central regulator FadR, were found in the LpxC and/or FtsH interactomes. Another protein associated with LpxC and FtsH was WaaH, a LPS-modifying enzyme. When overproduced, several members of the LpxC/FtsH interactomes were able to modulate LpxC proteolysis. Our results go beyond the previously established link between LPS and phospholipid biosynthesis and uncover a far-reaching network that controls LPS production by involving multiple enzymes in fatty acid metabolism, phospholipid biosynthesis and LPS modification.

## Introduction

Gram-negative bacteria like *Escherichia coli* are protected from external threats by two membranes mainly composed of PL. LPS on the surface of the outer membrane (OM) are exposed to the extracellular space in order to protect the cell from noxious compounds such as antibiotics or detergents (Nikaido, 2003; Bertani and Ruiz, 2018). LPS consist of three parts, the membrane-anchor lipid A, the connecting core oligosaccharide and the O-antigen (Raetz and Whitfield, 2002; Bertani and Ruiz, 2018). Biosynthesis of LPS starts with lipid A formation in the cytosol. LpxA catalyzes the first reaction of UDP-GlcNAc to the intermediate UDP-3-*O*-acyl-GlcNAc by using the acyl donor *R*-3-hydroxymyristoyl-ACP. The next modification step from UDP-3-*O*-acyl-GlcNAc to UDP-3-*O*-hydroxy-myristoyl-Glc is catalyzed by the essential deacetylase LpxC, and represents the first committed step of LPS biosynthesis (Young et al., 1995). FabZ catalyzes the first committed step of PL biosynthesis and competes with LpxA for the common precursor *R*-3-hydroxymyristoyl-ACP. To maintain a healthy balance between LPS and PL biosynthesis and to prevent toxic LPS accumulation in the periplasm, the amount of the LpxC enzyme is tightly controlled by the FtsH protease (Ogura et al., 1999). This activity renders the protease essential in *Escherichia coli*. An *ftsH* deletion strain only survives in the presence of hyperactive FabZ that counter-balances the high LpxC levels and prevents LPS overproduction (Ogura et al., 1999).

FtsH-mediated proteolysis of LpxC strictly follows the cellular demand. LpxC is stable in rapidly dividing cells when LPS biosynthesis is needed whereas the enzyme is degraded with a half-life around 10 min in slowly growing cells (Schäkermann et al., 2013). The molecular details of this differential proteolysis are largely unknown. Insights into this process hold promise for the development of antimicrobial compounds because both LpxC and FtsH are essential in a wide range of Gram-negative bacteria (Kalinin and Holl, 2016).

Proteolysis of LpxC by FtsH in *E. coli* requires a C-terminal tail with a minimal length of 20 amino acids and the sequence LAXXXXXAVLA (X = any amino acid) at its end (Führer et al., 2006). The degron is positioned in the flexible C-terminus of LpxC (Barb et al., 2007). It is necessary but not sufficient for FtsH-specific degradation. A C-terminal fusion of this sequence to the otherwise stable glutathione *S*-transferase (GST) renders it susceptible to degradation by FtsH and various other proteases suggesting the involvement of yet unknown interaction partners or internal structural motifs as determinants for FtsH specificity (Führer et al., 2007).

Degradation of many protease substrates requires the aid of accessory proteins, often called adaptor proteins (Sauer and Baker, 2011; Kuhlmann and Chien, 2017). Various proteins with an effect on LpxC degradation have been reported. Increased PL production by hyperactive FabZ stabilizes LpxC thereby restoring the balance between LPS and PL biosynthesis (Ogura et al., 1999; Schäkermann et al., 2013). Elevated levels of LapB (LPS assembly protein B, also known as YciM), which is essential under normal laboratory conditions, reduce the amount of LpxC and LPS. Conversely, LpxC is highly stabilized in a Δ*lapB* deletion strain causing cell death (Klein et al., 2014; Mahalakshmi et al., 2014). An additional effector of LpxC stability is the alarmone ppGpp, a signaling nucleotide synthesized by RelA and SpoT during the stringent response (Potrykus and Cashel, 2008). The stability of LpxC negatively correlates with the ppGpp level in the bacterial cell, i.e., the enzyme is stable during rapid growth when ppGpp levels are low, and it is degraded during slow growth when the ppGpp concentration is higher. This growth rate-dependent LpxC degradation is reversed in a strain lacking ppGpp. Here, LpxC is stable at slow and rapidly degraded at fast growth rates (Schäkermann et al., 2013). Most recently, it was reported that the outer membrane phospholipase PldA hydrolyses mis-localized PL and that the released fatty acids serve as second messenger to activate LPS biosynthesis. The fatty acids are converted to acyl-CoAs, which ultimately inhibit LpxC proteolysis (May and Silhavy, 2018). Computational modeling of the LPS biosynthesis pathway and experimental data support the hypothesis that lipid A disaccharide acts as feedback source and activates FtsH-dependent LpxC degradation (Emiola et al., 2014, 2016). How exactly FabZ, LapB, the nucleotide ppGpp, and the biosynthesis intermediates acyl-CoAs and lipid A disaccharide influence LpxC proteolysis is not yet understood.

The gaps in our knowledge on the regulation of this fundamentally important process in Gram-negative bacteria motivated us to search for more players involved in the homeostatic control network of LPS biosynthesis in *E. coli*. Since these players presumably interact with LpxC and/or FtsH either directly or in the framework of a large protein complex, we established an affinity purification-liquid chromatography tandem mass spectrometry (AP-LC-MS/MS)-based approach to identify the dynamic LpxC and FtsH interaction networks at different growth rates. Accurate quantification between different growth rates was achieved by a super-SILAC standard (Shenoy and Geiger, 2015). Using this robust quantification method, we successfully identified and validated several known and novel LpxC regulators providing new insights into the control network of LPS biosynthesis in *E. coli*.

## Materials and Methods

### Bacterial Strains and Plasmids

All bacterial strains and plasmids used in this study are listed in Supplementary Table S1. For *in vivo* degradation experiments, *E. coli* W3110 containing plasmids from the ASKA collection obtained from the National Bioresource Project, National Institute of Genetics, Japan were used (Kitagawa et al., 2005). When appropriate, antibiotics were used at following concentrations: ampicillin (Amp) 100 μg/mL, kanamycin (Kan) 50 μg/mL, chloramphenicol (Cm) 25 μg/mL, spectinomycin (Sp) 300 μg/mL.

### LpxC and FtsH Purification

*Escherichia coli* BL21 Δ*arg*/Δ*lys* cells were incubated in 800 mL LB medium at 180 rpm in a shaking water bath at 30°C (slow growth), 37°C (medium growth) or 40°C (fast growth) to an optical density (OD_580 nm_) of 0.5. Overproduction of Strep-LpxC (pBO113) or Strep-tag (pASK-IBA5+) were induced by adding 10 ng (30°C), 25 ng (37°C) or 40 ng (40°C) AHT for 30 min and overproduction of His_6_-MBP-FtsH (pMal-C-FtsH) or His_6_-MBP (pBO4811) were induced by adding 0.05 mM IPTG for 30 min at 30, 37, or 40°C. Cells were disrupted using the Constant Systems (two cycles, 40 kpsi) and centrifuged for 45 min at 13,200 rpm and 4°C. Strep-LpxC or Strep-tag were purified by Strep-tactin affinity purification (IBA Lifescience, manufacturer protocol) and His_6_-MBP-FtsH or His_6_-MBP by nickel-nitrilotriacetic acid (Ni-NTA) affinity purification as described previously (Westphal et al., 2012). Bradford assays were performed to determine the elution fraction with the highest amount of protein (Bradford, 1976).

### Preparation of the Super-SILAC Standard

*Escherichia coli* BL21 Δ*arg*/Δ*lys* were incubated in 500 mL Azure high-def media (Teknova) complemented with L-arginine: HCL (U-^13^C_6_, ^15^N_4_) (Arg_10_), L-lysine: 2HCL (^13^C_6_, ^15^N_2_) (Lys_8_) (Cambridge Isotope Laboratories) and 1% glucose at 30, 37, and 40°C until reaching an OD_580 nm_ of 0.5. Cells were harvested, pooled, disrupted by using the Constant System (two cycles, 40 kpsi) and centrifuged for 45 min at 13,200 rpm and 4°C. The protein concentration of the supernatant was determined by Bradford assay (Bradford, 1976). The total number of proteins and the labeling efficiency of Arg_10_ and Lys_8_ were determined after LC-MS/MS. The labeling efficiency of Arg_10_ and Lys_8_ was calculated by using following formula (with l = summed intensity of “light” peptides and h = summed intensity of “heavy” peptides):

$$100\%-\left(\frac{\Sigma \,\text{intensity l}}{\Sigma \,\text{intensity h}}\times 100\%\right)$$

### Liquid Chromatography and Mass Spectrometry

1.5 μg of purified Strep-LpxC or His_6_-MBP-FtsH were mixed with 1.5 μg of the super-SILAC standard. For Strep-tag or His_6_-MBP EV controls, the same volume as in the corresponding Strep-LpxC or His_6_-MBP-FtsH sample was used. Samples were concentrated on a Novex NuPage 4–12% gradient gel until the sample completely entered the gel. Proteins were visualized by colloidal Coomassie staining. Single protein bands were excised and washed with 40 μL of buffer A (10 mM NH_4_HCO_3_ in *A. dest.*) and 40 μL buffer B [buffer A in 50% (v/v) acetonitrile (ACN)]. Samples were evaporated in a vacuum concentrator. Dry gel pieces were digested using 5 μL of 2.0 μg/μL trypsin in 10 mM HCl and 55 μL of 100 mM NH_4_HCO_3_ overnight at 37°C. Peptides were extracted by incubating each sample in 20 μL 0.1% trifluoroacetic acid (TFA)/100% ACN (v/v = 1:1) in *A. dest*. twice for 10 min in an ultrasonic bath. The remaining ACN was removed by evaporation and 15 μL 0.1% TFA was added to the sample prior to LC-MS/MS analysis.

For peptide separation and identification an UltiMate^®^3000 RSLCnano HPLC system (Thermo Fisher Scientific) coupled to an Orbitrap Elite hybrid ion trap-Orbitrap mass spectrometer (Thermo Fisher Scientific) was used. 15 μL of the peptide sample was enriched and desalted on an Acclaim^®^PepMap^TM^ C18 μ-precolumn (5 μm particle size, 2 cm length, 100 μm ID, Thermo Fisher Scientific) using 95% solvent A [0.1% (v/v) TFA in *A. dest.*] and 5% solvent B [0.1% (v/v) TFA, 50% (v/v) ACN in *A. dest.*] with a flow rate of 30 μL/min for 7 min. Peptides were subsequently separated on an Acclaim^®^PepMap^TM^ C18 analytical column (2 μm particle size, 50 cm length, 75 μm ID, Thermo Fisher Scientific) using 95% solvent C [0.1% (v/v) formic acid (FA) in *A. dest.*] and 5% solvent D [0.1% (v/v) FA, 84% (v/v) ACN in *A. dest.*]. Peptides were eluted at a flow rate of 400 nL/min using a linear gradient of 5–40% solvent D over 120 min. Eluting peptides were injected in the mass spectrometer and a Fourier transform mass spectrometry (FTMS) scan in a mass/charge range between 300 and 2,000 with a resolution of 60,000 was performed. For MS/MS, the 20 most intense peptide ions (Top20 method) of the FTMS scan with a minimal signal intensity of 1,500 and a charge range >+2 were selected and fragmented by CID with a collision energy of 35%. The inclusion list size was set to 500 and the exclusion duration time to 35 s.

### Data Analysis

For quantitative analysis of high-resolution MS data, MaxQuant (Version 1.5.0.0, Max-Planck-Institute Martinsried) was used (Cox and Mann, 2008), which is based on the search algorithm Andromeda (Cox et al., 2011). All samples were processed together and parameters were set as follows: “Digestion Mode” = specific with enzyme Trypsin/P No “Fixed Modifications” were considered. “Variable modification” = oxidation of methionine, “Multiplicity” = 2, “Heavy labels” = Arg_10_ and Lys_8_, “Max. missed cleavages” = 2, “MS tolerance” = 20 ppm, “MS/MS tolerance” = 0.5 Da, “False discovery rate” (FDR) = 0.01, “Re-quantify” = checked, “Randomize” = decoy mode, “Minimal ratio count” = 1, “Match between runs” = unchecked, “iBAQ” = checked, “Include contaminants” = checked, “Cut peaks” = checked, “discard unmodified counterparts peptides” = unchecked. The *E. coli* reference proteome database (“ecoprot.lib” download from Uniprot, April, 4th, 2013, 4145 entries) was supplemented with the sequences of His_6_-MBP and Strep-tag and used to identify peptides and proteins. The taxonomy was set to “*E. coli.*” Only unique peptides were used for quantification. If not stated otherwise, default parameters in MaxQuant were used. Known contaminants were excluded. H/L ratios calculated by MaxQuant have been used (Cox et al., 2014).

### *In vivo* Degradation Using a Double Expression System

*Escherichia coli* W3110 cells containing a plasmid from the ASKA collection and the *lpxC* expression plasmid (pBO4804) were grown at 37°C and 180 rpm in a water bath shaker until an OD_580 nm_ of 0.5. The main culture was split into two subcultures. Expression of *lpxC* was induced in one subculture by adding 0.1% arabinose for 10 min and the gene of choice (ASKA collection) by adding 0.1–0.5 mM IPTG for 10–30 min. Only *lpxC* was overexpressed in the other subculture as a control. Translation was inhibited by adding 300 μg/mL Sp and samples were taken at different time points and frozen into liquid nitrogen. At least three biological replicates were analyzed.

### Sample Preparation and Immunodetection

Cell pellets were resuspended in TE-buffer (10 mM Tris-HCl, pH 8, 1 mM EDTA) in an OD_580 nm_-dependent manner (OD_580 nm_ of 1.0 = 100 μL TE-buffer) and protein sample buffer [final concentration: 2% SDS (w/v), 0.1% (w/v) bromophenol blue, 10% glycerol (v/v), 1% (v/v) β-mercaptoethanol, 50 mM Tris-HCl, pH 6.8] was added. The samples were incubated for 10 min at 95°C and centrifuged. Proteins in the supernatant were separated by SDS-PAGE and transferred on a nitrocellulose membrane according. LpxC was detected using the rabbit-α-Strep-tag-LpxC antibody followed by the goat-α-rabbit (H+L)-HRP conjugate (Bio-Rad) antibody. His_6_-tag fusion proteins were detected by using a Penta-His-HRP conjugate (Qiagen). Chemiluminescence signals were detected using Luminata Forte Western HRP substrate (Millipore) and the ChemiDoc^TM^ MP System (Bio-Rad). LpxC half-lives were calculated by using the software ImageJ (version 1.49^1^).

### Data Availability

The generated data have been deposited to the ProteomeXchange Consortium via the PRIDE partner repository (Vizcaíno et al., 2016) with the dataset identifier PXD006195.

## Results

The stability of LpxC and thereby the rate of LPS production is synchronized with the *E. coli* growth rate. It is conceivable that intracellular factors control the delicate balance of LpxC turnover by the FtsH protease and at least two scenarios are possible: adapter proteins might facilitate recognition of LpxC by FtsH at slow growth rates, e.g., by demasking crucial binding regions. Alternatively, recognition regions might be masked at high growth rates to avoid LpxC degradation. Therefore, we developed a protocol to capture proteins associated with the substrate LpxC and/or the responsible protease FtsH at different growth rates.

### Experimental Design

**FIGURE 1:**
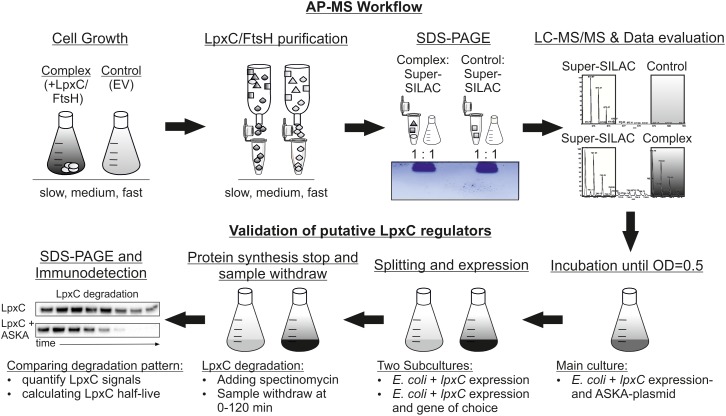
Experimental workflow to identify novel LpxC regulators by a super-SILAC quantification LC-MS approach. Plasmid-encoded Strep-LpxC or His_6_-MBP-FtsH (complex) proteins were overproduced at different growth rates (slow, medium and fast) in the *Escherichia coli* Δ*arg*/Δ*lys* strain with the corresponding EV (control). The internal super-SILAC standard contained labeled Arg_10_ and Lys_8_. After cell disruption, LpxC and FtsH variants were purified and mixed with equal amounts with the super-SILAC standard before purified proteins were identified using a short-gel LC-MS/MS approach. Statistically enriched proteins at single growth rates were validated using *in vivo* degradation experiments. An *E. coli* WT culture containing an *lpxC* and a gene of choice expression plasmid (ASKA-plasmid) were incubated until exponential growth-phase. The culture was split in two subcultures and *lpxC* and gene of choice expression were induced in the first subculture and only *lpxC* expression in the second as control. Translation was stopped by addition of spectinomycin and samples were taken between 0 and 120 min. After SDS-PAGE and immunodetection with an LpxC antibody half-life was calculated. AP-MS, affinity purification-mass spectrometry; EV, empty vector.

Aiming at the identification of modulators of LpxC stability, we used affinity-based protein purification combined with quantitative proteomics. Proteins associated with Strep-LpxC and MBP-FtsH were identified from cells harvested at different growth rates. The workflow is depicted in Figure 1. Strep-LpxC and His_6_-MBP-FtsH (and the corresponding control proteins Strep-tag and His_6_-MBP) were purified from *E. coli* cells after cultivation at different temperatures (30, 37, and 40°C) to adjust slow, medium and fast growth conditions. Successful purification was controlled by SDS-PAGE and Coomassie staining (Supplementary Figure S1). Three biological replicates of each culture resulted in a total number of 36 samples [9 Strep-tag (EV control), 9 Strep-LpxC, 9 His_6_-MBP (EV control) and 9 His_6_-MBP-FtsH] that were treated as described in Section “Materials and Methods.” For quantification, a heavy labeled super-SILAC standard was used as established previously (Arends et al., 2018). It derived from cells incubated at slow, medium and fast growth rates with a “heavy” labeling efficiency of 98.8%. 1345 proteins were identified in the pooled super-SILAC standard.

Proteins were considered as part of the interactomes using following criteria: (i) At least two “unique peptide” and a minimum of one “h/l ratio count” (“l intensity” > 0), (ii) a fold change (complex/Strep-tag or His_6_-MBP EV control) of at least “>2” and (iii) a *t*-test *p*-value of <0.05. If the protein was identified in 0–1 replicates in the EV control samples, the protein was categorized as “LpxC/FtsH only.”

Finally, the influence of a number of over-represented proteins on LpxC stability was examined by *in vivo* degradation experiments. Candidate proteins were overproduced and LpxC stability was monitored by SDS-PAGE, western transfer and immunodetection.

### Identification of the Dynamic LpxC and FtsH Interactome Networks

**FIGURE 2:**
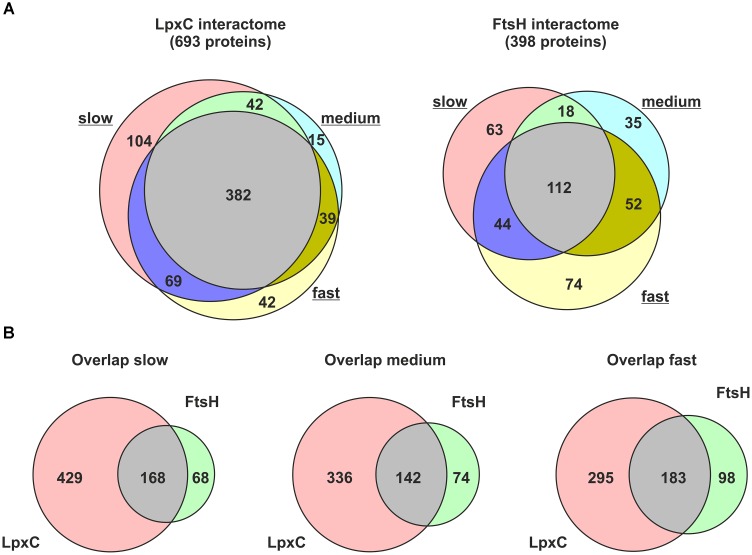
Proteins co-purified with Strep-LpxC or His_6_-MBP-FtsH at different growth-rates (slow, medium, and fast). Overlapping proteins between different growth-rates identified with Strep-LpxC or His_6_-MBP-FtsH are presented in **(A)**. The overlap between proteins enriched with Strep-LpxC and His_6_-MBP-FtsH at different growth-rates is visualized in **(B)**. More than 100 proteins overlapping between the LpxC and FtsH interactome networks at slow, medium or fast growth support a close connection between both networks.

Six hundred and ninety-three proteins were identified in the LpxC preparation; 382 of them were enriched under every growth condition. 398 proteins were pulled down with FtsH and 112 of them were enriched under every growth condition (Figure 2A and Supplementary Tables S2, S3 for LpxC and FtsH, respectively). This high number of putative LpxC and FtsH interaction partners demonstrated the sensitivity of the LC-MS/MS approach and revealed a substantial overlap of LpxC and FtsH interactors at all growth rates (Figure 2B). The identified proteins must not necessarily reflect direct protein-protein contacts but are suggestive of highly dynamic and complex LpxC and FtsH interactome networks. The presence of various known LpxC and FtsH interactors shows that our dataset includes direct protein-protein interactions (Table 1). FtsH, PyrH, GroEL, and SucB were previously reported to interact with LpxC. The FtsH dataset includes the known modulators HflK and HflC as well as several FtsH substrates (DadA, PpiD, IscS, and SecD).

**Table 1:** Known LpxC and FtsH interactors identified by the super-SILAC AP-MS approach.

Known LpxC interactors				Reference

	30°C (slow growth)	37°C (medium growth)	40°C (fast growth)	
FtsH	53.6	172.7	128.7	Ogura et al., 1999
PyrH	22.3	6.4	45.0	Butland et al., 2005
GroEL	15.9	59.8	43.5	Arifuzzaman et al., 2006
SucB	96.5	293.3	133.0	Arifuzzaman et al., 2006
**Known FtsH interactors or substrates^∗^**				
HflC	4.2	3.0	3.4	Kihara et al., 1996
HflK	4.6	4.5	5.5	Kihara et al., 1996
LpxA	2.6		3.7	Butland et al., 2005
Tig		18.4	12.3	Arifuzzaman et al., 2006
PhoP			3.1	Arifuzzaman et al., 2006
DadA^∗^	FtsH only		5.8	Bittner et al., 2015
PpiD^∗^	5.9		4.3	Bittner et al., 2015
IscS^∗^	4.2			Westphal et al., 2012
SecD^∗^		2.2		Arends et al., 2016


*FtsH substrates are marked with an asterisk. Enrichment factors (LpxC/EV and FtsH/EV) are given for the cultures grown at 30, 37, and 40 °C. The significance threshold was set at 0.05 (Student’s t-test).*

**FIGURE 3:**
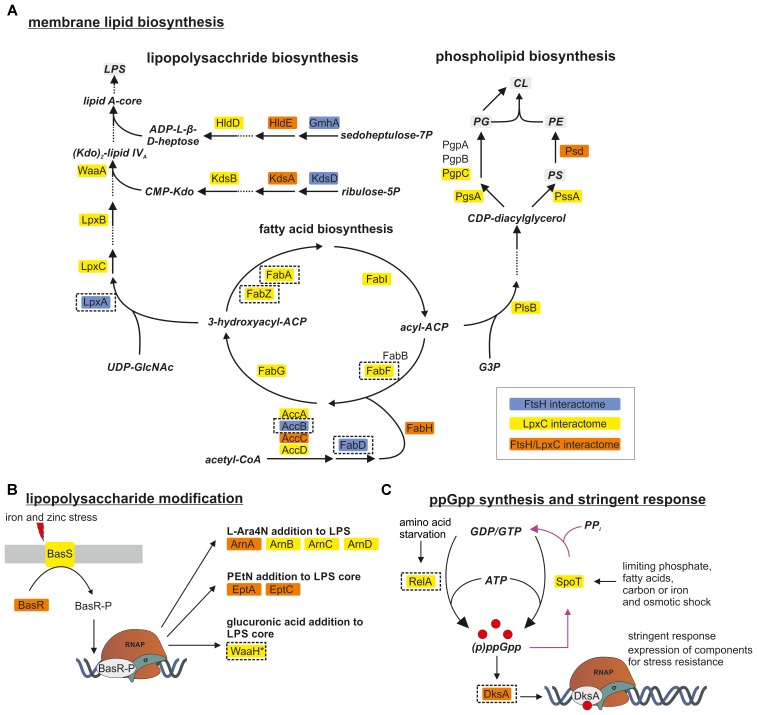
FtsH and LpxC are strongly associated with proteins involved in membrane lipid biosynthesis **(A)**, LPS modification **(B)** and synthesis of the alarmone ppGpp **(C)**. LPS and PL biosynthesis are closely connected since both need products from FAB. Proteins identified in one of the FtsH, LpxC or both interactomes are marked in blue, yellow and orange, respectively. Proteins selected for downstream experiments are boxed with dashed lines. For a better overview, not all proteins of the illustrated pathways are shown (dashed arrows). CoA, coenzyme A; ACP, acyl carrier protein; UDP-GlcNAc, UDP-N-acetylglucosamine; Kdo, 2-keto-3-desoxy-octonat; ADP-L-β-D-heptose, ADP-L-glycero-β-D-*manno*-heptose; G3P, glycerol-3-phosphate; PG, phosphatidylglycerol; PC, phosphatidylcholine; PS, phosphatidylserine; CL, cardiolipin; PE/PEtN, phosphatidylethanolamine; L-Ara4N, 4-amino-4-deoxy-L-arabinose.

One of the most striking findings of our analysis was that many proteins involved in membrane lipid biosynthesis were associated with LpxC (Figure 3A and Table 2) suggesting a functional supercomplex coordinating fatty acid, PL and LPS biosynthesis. Among the enzymes involved in LPS production was WaaA (KdtA), which acts several steps downstream of LpxC and is another substrate of the FtsH protease (Katz and Ron, 2008). Remarkably, almost all proteins of the fatty acid elongation cycle were found in the LpxC interactome. It is conceivable that these proteins were recruited to the complex via FabZ, a reported interactor of FAB enzymes. FadR, the master regulator of FAB and degradation was also present in this supercomplex (Table 2). These findings are consistent with the previously reported intricate crosstalk between fatty acid and LPS biosynthesis (Emiola et al., 2016). Moreover, we identified many proteins involved with the modification of LPS in response to environmental stress conditions like elevated iron or zinc concentrations (Figure 3B and Table 2) (Nagasawa et al., 1993; Hagiwara et al., 2004; Lee et al., 2005). On transcriptional level, they are regulated by the BasSR two-component system, which was also identified in the LpxC interactome. The association of several proteins involved in membrane lipid production with FtsH is consistent with a role of the protease in LPS biosynthesis. In light of the previously reported effect of the signaling nucleotide ppGpp on LpxC proteolysis, it is noteworthy that the ppGpp synthases RelA and SpoT and the alarmone binding transcriptional factor DksA were found in the LpxC network (Figure 3C and Table 2).

**Table 2:** List of proteins involved in membrane lipid biosynthesis, LPS modification and synthesis of the alarmone ppGpp which were identified in the FtsH or LpxC interactomes.

		LpxC 30°C		LpxC 37°C		LpxC 40°C		FtsH 30°C		FtsH 37°C		FtsH 40°C	
		
		Ratio	*p*-value	Ratio	*p*-value	Ratio	*p*-value	Ratio	*p*-value	Ratio	*p*-value	Ratio	*p*-value
**Lipopolysaccharide biosynthesis**								
**LpxA**	Lipid A synthesis, UDP-*N*-acetylglucosamine acyltransferase							2.58	0.020			3.71	0.001
LpxB	Lipid A disaccharide synthase			LpxC only	LpxC only								
WaaA	3-deoxy-D-manno-octulosonate-lipid A transferase	LpxC only	LpxC only	LpxC only	LpxC only	LpxC only	LpxC only						
KdsD	D-arabinose 5-phosphate isomerase											5.38	0.016
KdsA	3-deoxy-D-manno-octulosonate 8-phosphate synthase	LpxC only	LpxC only							5.39	0.000		
KdsB	3-deoxy-manno-octulosonate cytidylyltransferase	20.37	0.001										
GmhA	Phosphoheptose isomerase											3.82	0.001
HldE	Heptose 7-P kinase/heptose 1-P adenyltransferase	41.50	0.001	21.48	0.000	28.54	0.000	7.82	0.016	3.57	0.009	5.95	0.002
HldD	ADP-L-glycero-D-manno-heptose-6-epimerase	10.57	0.006	4.83	0.019	2.70	0.002						
**LapB**	Lipopolysaccharide assembly protein B	LpxC only	LpxC only	LpxC only	LpxC only	LpxC only	LpxC only						
**Phospholipid biosynthesis**								
PlsB	Glycerol-3-phosphate acyltransferase	33.48	0.000	43.71	0.003	69.98	0.000						
PssA	Phosphatidylserine synthase	7.16	0.005			10.11	0.003						
PgsA	Phosphatidylglycerophosphate synthase	LpxC only	LpxC only										
Psd	Phosphatidylserine decarboxylase, phospholipid biosynthesis	7.64	0.001	8.58	0.000	LpxC only	LpxC only	FtsH only	FtsH only	FtsH only	FtsH only		
PgpC	Phosphatidylglycerophosphatase C, membrane bound	LpxC only	LpxC only										
**Fatty acid biosynthesis**								
AccA	Acetyl-CoA carboxylase, carboxyltransferase alpha subunit	52.05	0.000	16.00	0.001	16.94	0.001						
**AccB**	Acetyl-CoA carboxylase, biotin carboxyl carrier protein									3.40	0.014	3.00	0.016
AccC	Acetyl-CoA carboxylase, biotin carboxylase (BC) subunit	30.40	0.000	49.89	0.001	16.01	0.000			3.29	0.022	3.44	0.003
AccD	Acetyl-CoA carboxylase, carboxyltransferase beta subunit	11.83	0.000	10.79	0.011	27.35	0.025						
**FabD**	Malonyl-CoA-acyl carrier protein transacylase							8.41	0.017				
FabH	Beta-Ketoacyl-ACP synthase III	43.09	0.000	19.42	0.000	13.49	0.000					4.05	0.001
FabG	Beta-Ketoacyl-ACP reductase	153.60	0.001	62.66	0.000	88.41	0.003						
**FabF**	Beta-Ketoacyl-ACP synthase II	6.28	0.009	13.00	0.012	11.32	0.001						
**FabZ**	3R-hydroxymyristoyl acyl carrier protein (ACP) dehydratase	5.03	0.007	LpxC only	LpxC only								
**FabA**	3R-3-hydroxydecanoyl acyl carrier					3.16	0.012						
FabI	Enoyl-ACP reductase, NADH dependent	19.71	0.005	6.51	0.005	7.75	0.002						
**FadR**	Repressor/activator for fatty acid metabolism regulon	LpxC only	LpxC only	LpxC only	LpxC only								
**Lipopolysaccharide modification**								
BasS	Histidine protein kinase sensor for Lipid A modification genes	LpxC only	LpxC only	LpxC only	LpxC only	LpxC only	LpxC only						
BasR	Response regulator for Lipid A modification genes	LpxC only	LpxC only					5.60	0.022				
EptA	Lipid A phosphoethanolamine transferase	LpxC only	LpxC only			LpxC only	LpxC only	FtsH only	FtsH only	FtsH only	FtsH only	FtsH only	FtsH only
EptC	LPS heptose I phosphoethanolamine transferase	191.97	0.000	76.06	0.002	74.39	0.000			5.20	0.031	3.67	0.017
ArnA	UDP-glucuronate dehydrogenase and UDP-ara4N formyltransferase	82.77	0.000	57.11	0.000	23.93	0.000					3.25	0.012
ArnB	UDP-4-amino-4-deoxy-L-arabinose			19.66	0.024	14.68	0.022						
ArnC	Undecaprenyl phosphate-aminoarabinose synthase	LpxC only	LpxC only	120.43	0.006	LpxC only	LpxC only						
ArnD	Undecaprenyl phosphate-aminoarabinose deformylase	LpxC only	LpxC only			LpxC only	LpxC only						
**WaaH**	LPS (HepIII)-glucuronic acid glycosyltransferase	LpxC only^∗^	LpxC only^∗^					FtsH only^∗^	FtsH only^∗^				
**ppGpp synthesis and stringent response**								
**RelA**	ATP:GTP 3-pyrophosphotransferase, ppGpp synthase I	4.67	0.005	8.41	0.012	4.10	0.005						
SpoT	ppGpp 3-pyrophosphohydrolase and ppGpp synthase II	13.07	0.000	19.48	0.001								
**DksA**	RNAP-binding protein modulating ppGpp and iNTP regulation	2.86	0.017			LpxC only	LpxC only	2.91	0.030				
**Miscellaneous functions**								
**FadB**	Multifunctional fatty acid oxidation complex subunit alpha			LpxC only	LpxC only	LpxC only	LpxC only						
**PyrH**	Uridylate kinase	22.30	0.001	6.43	0.000	45.03	0.036						
**LamB**	Maltoporin, maltose high-affinity uptake system	LpxC only	LpxC only					FtsH only	FtsH only				


*Enrichment factors (LpxC/EV and FtsH/EV) are given for the cultures grown at 30, 37, and 40°C. The significance threshold was set at 0.05 (Student’s t-test). Proteins selected for downstream experiments are marked in bold. WaaH is marked with an asterisk since it was identified with one unique peptide.*

### Identification of Putative LpxC Regulators

LpxC or FtsH interactors with an impact on LPS biosynthesis should affect the half-life of LpxC when they are overproduced. To analyze whether some of the proteins found in these large networks modulate LpxC stability, we selected several candidates for subsequent validation experiments. Because a putative modulator can in principle interact with LpxC and/or FtsH, we selected proteins found in the LpxC (LapB, FabF, FabA, FabZ, FadR, RelA, FadB, and PyrH), FtsH (LpxA, AccB, and FabD) or both interactomes (WaaH, DksA, and LamB). We paid particular attention to candidates that preferentially interact with LpxC and/or FtsH under slow (e.g., WaaH and FabD), fast (FabA) or all (e.g., LapB and RelA) growth rates.

**FIGURE 4:**
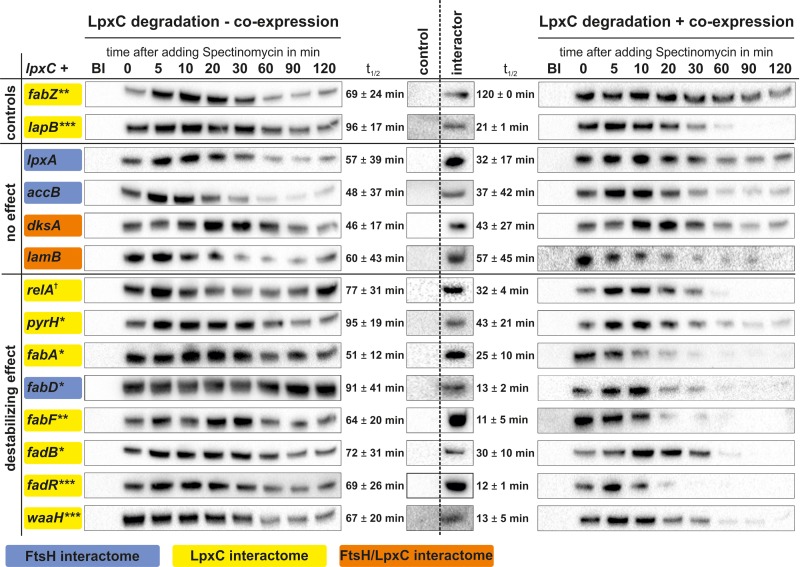
*In vivo* LpxC degradation experiments using a double expression system for identification of factors influencing LpxC turnover. Expression of *lpxC* **(left)** or *lpxC* and a gene of choice **(right)** was induced in exponential growth phase and translation was stopped by adding spectinomycin. Samples were taken before (BI) and 0–120 min after stopping translation. Overproduction of putative LpxC interactors compared to the control was verified by SDS-PAGE followed by immunodetection **(middle)**. After SDS-PAGE and immunodetection LpxC signals were quantified and half-lives calculated. Overexpression of *fabZ* led to stabilized LpxC (higher LpxC t_1/2_ after *fabZ* expression) and *lapB* to destabilized LpxC (lower LpxC t_1/2_ after *lapB* expression) (controls). Gene products, which did not influence LpxC degradation (no effect) and genes which caused rapid LpxC degradation (destabilized effect), are shown. Standard deviations were calculated from at least three biological replicates (Supplementary Table S4). ^†^*p* ≤ 0.15, ^∗^*p* ≤ 0.10, ^∗∗^*p* ≤ 0.05, ^∗∗∗^*p* ≤ 0.01.

Candidate proteins (boxed in Figure 3 and in bold in Table 2) were subjected to *in vivo* LpxC degradation experiments (Figure 1). Corresponding expression plasmids were obtained from the ASKA collection and the production of the proteins was monitored by Western blot analysis. The stability of plasmid-encoded LpxC was determined in the absence or presence of overproduced amounts of the putative regulator proteins (Figure 4).

The known LpxC regulators FabZ and LapB, which have opposing effects on LpxC stability, served as controls. As expected from previous observations (Mohan et al., 1994; Ogura et al., 1999; Schäkermann et al., 2013), overexpression of *fabZ* led to an increased stability of LpxC. Also as expected (Klein et al., 2014; Mahalakshmi et al., 2014), an accelerated LpxC degradation was observed after overproducing LapB. These results demonstrate that the two-plasmid system is suited for testing candidate proteins that might influence LpxC degradation.

Overexpression of *lpxA, accB*, *dksA*, or *lamB* had no or only minor effects on LpxC stability (Figure 4). Previously, the key enzyme for ppGpp production, RelA, was shown to effect LpxC degradation as LpxC was stabilized under slow-growing conditions (when it should be degraded) in the *relA* mutant (Schäkermann et al., 2013). Accordingly, the opposite effect was observed when RelA was overproduced, which led to destabilized LpxC compared to the control. Overproduction of the previously described LpxC interactor PyrH (Butland et al., 2005), an essential enzyme for *de novo* synthesis of pyrimidine nucleotides, also destabilized LpxC. Most interestingly, the overproduction of several proteins involved in FAB or degradation, namely FabA, FabD, FabF, FadB, and FadR, destabilized LpxC suggesting a close link between LPS biosynthesis and fatty acid homeostasis. Another interesting modulator of LpxC stability is the glucuronic acid transferase WaaH, which is involved in the modification of the LPS core oligosaccharide under stress conditions (Klein et al., 2013). It was identified as LpxC and FtsH interactor exclusively in samples from slow-growing cultures, i.e., under conditions when LpxC is rapidly degraded (Table 2). Consistent with this condition-dependent co-purification, WaaH destabilized LpxC and reduced its half-life from 47 min under control conditions to 21 min when it was overproduced (Figure 4).

## Discussion

The spatial and temporal control of LPS biosynthesis is vitally important for Gram-negative bacteria and several enzymes in this process are essential in *E. coli* making them attractive antimicrobial targets. The rate of LPS production is determined by the intracellular level of the key enzyme LpxC, which is adjusted by the FtsH protease (Ogura et al., 1999; Führer et al., 2006). Tipping the balance toward too much or too little LpxC enzyme, results in deleterious over- or under-accumulation of LPS. Although previous studies revealed several factors involved in the coordination of this delicate process, they also revealed gaps in our knowledge, which motivated us to search for novel LpxC and FtsH modulators. Our super-SILAC LC-MS/MS approach was successful in the identification of proteins with a profound impact on LpxC stability (Figure 4). The identification of several known LpxC and FtsH interaction partners such as HflKC and LapB (Kihara et al., 1996; Klein et al., 2014) further validated Super-SILAC as a suitable quantification technique for *E. coli*. The advantage of such a super-SILAC standard is the universal applicability for several independent experimental designs. In fact we recently took advantage of the same super-SILAC standard for the identification of substrates of the Lon protease in *E. coli* (Arends et al., 2018).

### Known and Novel Effectors for LPS Biosynthesis

To discover proteins that influence the growth rate-dependent LpxC proteolysis by FtsH, the effect of 14 LpxC/FtsH associated proteins on LpxC stability was analyzed by *in vivo* degradation experiments. Overproduction of nine proteins (LapB, FabA, FabD, FabF, FadB, FadR, PyrH, RelA, and WaaH) stimulated LpxC degradation whereas overproduction of FabZ let to LpxC stabilization. The influence of the dehydratase FabZ, the LPS assembly protein LapB, and the alarmone ppGpp on LpxC proteolysis had already been demonstrated in previous experiments (Ogura et al., 1999; Schäkermann et al., 2013; Klein et al., 2014; Mahalakshmi et al., 2014). The effects of overproduced amounts of FabZ, LapB and the ppGpp synthase RelA were fully consistent with these reports and thus validated the double expression system as suitable approach for testing LpxC regulators.

Interestingly, the known LpxC regulators co-purified with LpxC suggesting either a direct interaction or their presence in a large protein–protein network. FabZ was overrepresented at slow and medium growth rates in the LpxC interactome. Overproduction of FabZ directs 3-hydroxyacyl-ACP, the common precursor of PL and LPS biosynthesis, away from the LpxC enzyme, which results in LpxC stabilization supporting an intimate crosstalk between LPS and PL biosynthesis. The essential metal-binding protein LapB interacts with a number of proteins involved in the LPS pathway like WaaC, FtsH, the chaperones DnaJ and DnaK (Klein et al., 2014). LapB regulates LpxC degradation, is conserved in many enterobacteria, and the absence of this protein caused elongated *E. coli* cells, much like LpxC accumulation (Mahalakshmi et al., 2014). LapB was suggested to be a positive LpxC regulator, which mediates FtsH-specific degradation and orchestrates the assembly of LPS biosynthesis enzymes at the inner membrane (Klein et al., 2014; Mahalakshmi et al., 2014; Nicolaes et al., 2014). We did not identify LapB in the FtsH interactome but in the LpxC interactome network under all conditions, and the stimulatory effect on LpxC degradation (Figure 4) is fully consistent with a function as adaptor protein.

A putative novel LpxC regulator is the essential UMP kinase PyrH, which is involved in the *de novo* synthesis of pyrimidines (Serina et al., 1995). LpxC was previously identified as PyrH interaction partner in a global protein-protein interaction study (Butland et al., 2005). PyrH mutants with reduced GTP activity are SDS sensitive, a phenotype also observed when LpxC is stabilized or overproduced (Yamanaka et al., 1992; Führer et al., 2006). PyrH was highly enriched with LpxC at all growth rates. The half-life of LpxC decreased with *pyrH* co-expression suggesting a dual function of PyrH in LPS biosynthesis presumably by regulating the pool of UDP-3-*O*-acyl-GlcNAc and *de novo* biosynthesis of pyrimidine nucleotides (Serina et al., 1995). The precursor UDP-GlcNAc is also used for peptidoglycan biosynthesis and competition for the same substrate might influence LpxC stability. In support of this assumption, we identified several proteins involved in cell wall biosynthesis in the LpxC interactome (Supplementary Table S2).

### Crosstalk Between FAB, FAD, and LPS Biosynthesis

Computational modeling of LPS biosynthesis revealed that regulation of LpxC stability is more complex than simple competition for 3-hydroxyacyl-ACP by LpxC and FabZ (Emiola et al., 2016) and our data agree with this model to a large extent. One of the most compelling results of our study is that FadR and various enzymes from the FAB and FAD pathways were able to modulate the cellular LpxC level. Except for FabZ (LpxC stabilator; see above) and AccB (no effect) all tested proteins from this metabolic pathway accelerated LpxC degradation. The transcription factor FadR is responsible for the regulation of several genes crucial for FAB or FAD (DiRusso et al., 1992; Xu et al., 2001; My et al., 2015). The activity of FadR is dependent on the availability of LCFA bound to acyl-CoA. Binding of the FadR homodimer to promoter regions as an apoprotein activates transcription of genes involved in the FAB and represses genes associated with the FAD (Raman et al., 1997). Binding of LCFA-containing acyl-CoA to FadR activates the FAD and shuts down the FAB pathway (Cronan and Subrahmanyam, 1998). LCFA are produced in the FAB and degraded in the FAD pathway (Henry and Cronan, 1991; DiRusso et al., 1999; My et al., 2015). We found that overproduction of FadR destabilized LpxC at slow, medium and fast growth rates (Figure 4 and data not shown). As the central switch between FAB and FAD, FadR presumably regulates LpxC degradation via the amount of Acyl-CoA, which is crucial for LPS and PL biosynthesis. Endogenous LCFA amounts are probably too low to interact with the overproduced transcription factor. Therefore, FadR is expected to bind the DNA in its active form as a dimer leading to expression of genes crucial for the FAB leading to overproduction of fatty acids (Henry and Cronan, 1991, 1992; DiRusso et al., 1992; Zhang et al., 2012). The FadR regulon includes the *fabHDG* operon and *fabA*, *fabB*, *fabI* (My et al., 2013, 2015), which might explain that overproduction of FabA, FabD, and FabF also destabilized LpxC. In this context it is also interesting that ppGpp was shown to reduce expression of *fadR* on transcriptional level, which might be one of the reasons why the signaling nucleotide ppGpp has a profound effect on LpxC stability (My et al., 2013; Schäkermann et al., 2013).

Our results support the idea of an intricate crosstalk between FAB und LPS biosynthesis. It has been reported that an increased substrate flux into the saturated FAB pathway accelerates LpxC degradation (Emiola et al., 2016). High amounts of saturated fatty acids were suggested to repress LpxK, an enzyme acting downstream of LpxC in the lipid A biosynthesis pathway (Emiola et al., 2014, 2016). This leads to an accumulation of the LpxK substrate lipid A disaccharide, which was postulated to activate FtsH resulting in faster LpxC degradation (Emiola et al., 2014) similar to the situation during FadR overproduction. A temperature-sensitive *fabI* mutant grown at non-permissive temperatures increased substrate flux into unsaturated FAB resulting in highly stabilized LpxC. Moreover, consistent with our results it was reported that overproduction of FabA, which stimulates saturated fatty acid production reduces LpxC stability (Emiola et al., 2016). Production of FabF and FabD, which are involved in fatty acid elongation, also stimulated LpxC degradation. FabF catalyzes the conversion of palmitoleate to *cis*-vaccenate (Garwin et al., 1980) and FabD produces malonyl-ACP and has a positive regulatory effect on *fabZ* (Magnuson et al., 1992).

The previously reported toxic effect of increased synthesis of LCFA in *E. coli* (Lennen et al., 2011) might also relate to LPS biosynthesis. Addition of LCFA palmitoyl-CoA to *E. coli* cells *in vitro* was shown to have a stabilizing effect on LpxC and might inactivate FadR and the enzymes taking part in FAB biosynthesis, which would lead to a dysregulation of LPS biosynthesis (Emiola et al., 2016). Furthermore, an interaction of LpxC with LCFA was suggested, which might exacerbate the toxic growth defect after increased LCFA addition (Lennen et al., 2011; Emiola et al., 2016). LpxC was also destabilized after FadB overproduction. Together with FadA, FadB is part of the aerobic fatty acid complex catalyzing the β-oxidation of LCFA (Yang et al., 1991; Yang and Elzinga, 1993). Overproduction of FadB might result in a higher turnover of LCFA to short-chain fatty acids (SCFA) and acetyl-CoA. As LPS biosynthesis precursor molecules are based on LCFA, the amount of LpxC, the key enzyme of the LPS biosynthesis, is decreased.

LpxC degradation in *in vitro* degradation experiments using the lysate of an *ftsH* deletion strain after addition of palmitic acid suggested the participation of another metalloprotease in the degradation of LpxC, which might be induced by LCFA (Emiola et al., 2016). Interestingly, the zinc metalloprotease PrlC was enriched with LpxC (19-, 10-, or 8-fold at 30, 37, or 42°C, respectively) making it a very promising candidate as secondary protease in LpxC degradation (Supplementary Table S2).

### The Putative Dual Function of WaaH in LPS Biosynthesis

The glucuronic acid transferase WaaH modifies core oligosaccharides of LPS under phosphate, Fe^3+^ or Zn^2+^ limited conditions and in stationary growth phase (Klein et al., 2013). Expression of *waaH* is induced by the BasS/R and PhoR/B two-component systems (Baek and Lee, 2006; Froelich et al., 2006). WaaH modifies LPS by catalyzing the addition of glucuronic acid to the third heptose of the inner core oligosaccharide. Our results suggest a second function of WaaH as putative FtsH and/or LpxC modulator. LpxC is typically degraded under slow growth conditions, like in stationary phase, i.e., when *waaH* is expressed. Accordingly, WaaH was identified exclusively at slow growth rates in the LpxC and FtsH interactome networks. Recently, WaaH was found to be membrane-associated (Klein et al., 2013), which might further support an adapter function in recruiting LpxC to the membrane-anchored FtsH protease.

Remarkably, we identified several other LPS-modifying enzymes in the LpxC interactomes. All of them are under the control of the BasS/R system. The PmrA/B system of *Salmonella* (a homolog of BasS/R) is known to regulate genes involved remodeling of the outer membrane to control the permeability (Groisman, 2001; Herrera et al., 2010). Maybe the overproduction of proteins from this regulon leads to outer membrane perturbations and the cell shut down LPS biosynthesis to avoid an unhealthy accumulation of modified LPS. That outer membrane perturbations can affect LpxC stability was recently shown as mislocalized PL in the outer membrane stimulate LPS biosynthesis by stabilization of LpxC (May and Silhavy, 2018).

## Conclusion

Although molecular details of the intricate network remain to be fully explored, our super-SILAC-based study provided unprecedented insights into a far-reaching crosstalk between LPS, PL and fatty acid metabolism in *E. coli*. A tight coordination of inner and outer membrane biosynthesis via FabZ and LpxC was also recently reported in *Klebsiella pneumoniae* (Mostafavi et al., 2018). Our results showed that disturbing the balance in these pathways by overproduction of individual proteins from different branches of membrane lipid biosynthesis can modulate the rate of LpxC degradation, and hence LPS production. At least four of the proteins involved in this network (LpxC, FtsH, LapB, and PyrH) are essential and might serve as “Achilles heals” for the development of antimicrobial strategies against Gram-negative bacteria.

## Author Contributions

NT, JA, HM, KM, and FN performed the study design. NT and JA carried out the experiments. NT, JA, CL, and KB performed the data analysis. NT, JA, KM, and FN wrote the manuscript.

## Conflict of Interest Statement

The authors declare that the research was conducted in the absence of any commercial or financial relationships that could be construed as a potential conflict of interest.

## Footnotes

1 http://imagej.nih.gov/ij

## Abbreviations

ACP: acyl carrier protein
AP-MS: affinity purification-mass spectrometry
EV: empty vector, FAB, fatty acid biosynthesis
FAD: fatty acid degradation
LCFA: long-chain fatty acid
LC-MS/MS: liquid chromatography tandem mass spectrometry
LPS: lipopolysaccharide
MBP: maltose binding protein
PL: phospholipid
ppGpp: guanosine tetraphosphate
SILAC: stable isotope labeling with amino acids in cell culture
