# Supplementary material for: Intricate Crosstalk Between Lipopolysaccharide, Phospholipid and Fatty Acid Metabolism in Escherichia coli Modulates Proteolysis of LpxC
Source: Front Microbiol. 2019 Jan 14;9:3285. doi: 10.3389/fmicb.2018.03285 (PMC6339880; doi:10.3389/fmicb.2018.03285)
Supplement: Supplementary file 1 [file Table_1.DOCX]

**SUPPLEMENTARY TABLE 1.** Bacterial strains and plasmids

| Strains or plasmids | Properties | Reference or source |
| --- | --- | --- |
| *E. coli strains* |  |  |
| BL21 Δ*arg*/Δ*lys* | F-, *ompT, gal (dcm) (lon)*, *hsdSB (rB-mB-)*, λ [DE3], Δ*arg*/Δ*lys* | ([Matic et al. 2011](#_ENREF_5)) |
| W3110 | F^-^, IN (*rrnD-rrnE*)1 | ([Bachmann 1972](#_ENREF_1)) |
| *plasmids* |  |  |
| pASK-IBA5+ | Amp^r^, *tetR*, P/O_tet_, encoding Strep-tag | IBA lifescience |
| pMal-C-FtsH | Amp^r^, P_lac_, *lacI*; pMal-C derivate encoding His_6_-MBP-FtsH | ([Katz and Ron 2008](#_ENREF_3)) |
| pCA24N derivate | Cm^R^, P_T5/lac_, *lacI^q^, gfp-* | ([Kitagawa et al. 2005](#_ENREF_4)) |
| pBO113 | pASK-IBA5+ derivate encoding Strep-LpxC | ([Führer, Langklotz, and Narberhaus 2006](#_ENREF_2)) |
| pBO4804 | Amp^r^, P_BAD_, *araC,* pBAD30 derivate encoding LpxC | this study |
| pBO4811 | pMal-C-FtsH derivate encoding His_6_-MBP | this study |

**References**

Bachmann, B. J. 1972. 'Pedigrees of some mutant strains of *Escherichia coli* K-12', *Bacteriol. Rev.*, 36: 525–57.

Führer, F., S. Langklotz, and F. Narberhaus. 2006. 'The C-terminal end of LpxC is required for degradation by the FtsH protease', *Mol. Microbiol.*, 59: 1025-36.

Katz, C., and E. Z. Ron. 2008. 'Dual role of FtsH in regulating lipopolysaccharide biosynthesis in *Escherichia coli*', *J. Bacteriol.*, 190: 7117–22.

Kitagawa, M., T. Ara, M. Arifuzzaman, T. Ioka-Nakamichi, E. Inamoto, H. Toyonaga, and H. Mori. 2005. 'Complete set of ORF clones of *Escherichia coli* ASKA library (a complete set of *E. coli* K-12 ORF archive): unique resources for biological research', *DNA Res.*, 12: 291-99.

Matic, I., E. G. Jaffray, S. K. Oxenham, M. J. Groves, C. L. R. Barratt, S. Tauro, N. R. Stanley-Wall, and R. T. Hay. 2011. 'Absolute SILAC-compatible expression strain allows Sumo-2 copy number determination in clinical samples', *J. Proteome Res.*, 10: 4869–75.
