# Supplementary material for: Intricate Crosstalk Between Lipopolysaccharide, Phospholipid and Fatty Acid Metabolism in Escherichia coli Modulates Proteolysis of LpxC
Source: Front Microbiol. 2019 Jan 14;9:3285. doi: 10.3389/fmicb.2018.03285 (PMC6339880; doi:10.3389/fmicb.2018.03285)
Supplement: Supplementary file 4 [file Table_4.docx]

**Supplementary Table S4**

Summary of *in vivo* LpxC degradation experiments using a double expression system for identification of factors influencing LpxC turnover. Mean half-life (t_1/2_) of LpxC and standard deviation are shown for experiments without (- co-expression) and after overproduction (+ co-expression) of the given proteins. Ratios were calculated using mean values. Significant differences between mean t_1/2_ was tested with a two-sided Student’s t-test.

|  |  | **t_1/2_ LpxC [min]** | |  | |
| --- | --- | --- | --- | --- | --- |
| **protein** | **# of experiments** | **- co-expression** | **+ co-expression** | **ratio (+/-)** | **p-value** |
| **controls** | | | | | |
| FabZ | 3 | 69 ± 24 | 120 ± 0 | 1.74 | 0.040 |
| LapB | 3 | 96 ± 17 | 21 ± 1 | 0.22 | 0.003 |
| **no effect** | | | | | |
| LpxA | 4 | 57 ± 39 | 32 ± 17 | 0.56 | 0.355 |
| AccB | 5 | 48 ± 37 | 37 ± 42 | 0.77 | 0.705 |
| DksA | 3 | 46 ± 17 | 43 ± 27 | 0.94 | 0.912 |
| LamB | 3 | 60 ± 43 | 57 ± 45 | 0.95 | 0.949 |
| **destabilizing effect** | | | | | |
| RelA | 3 | 77 ± 31 | 32 ± 4 | 0.42 | 0.111 |
| PyrH | 3 | 95 ± 19 | 43 ± 21 | 0.45 | 0.057 |
| FabA | 3 | 51 ± 12 | 25 ± 10 | 0.50 | 0.081 |
| FabD | 3 | 91 ± 41 | 13 ± 2 | 0.15 | 0.058 |
| FabF | 3 | 64 ± 20 | 11 ± 5 | 0.17 | 0.023 |
| FadB | 4 | 72 ± 31 | 30 ± 10 | 0.42 | 0.069 |
| FadR | 6 | 69 ± 26 | 12 ± 1 | 0.17 | 0.004 |
| WaaH | 4 | 67 ± 20 | 13 ± 5 | 0.19 | 0.004 |
