# Supplementary material for: Intricate Crosstalk Between Lipopolysaccharide, Phospholipid and Fatty Acid Metabolism in Escherichia coli Modulates Proteolysis of LpxC
Source: Front Microbiol. 2019 Jan 14;9:3285. doi: 10.3389/fmicb.2018.03285 (PMC6339880; doi:10.3389/fmicb.2018.03285)

**Supplementary Figure S1. Coomassie gels after Strep-LpxC, His_6_-MBP-FtsH and the corresponding control purifications from cells carrying the empty vector (VV).** Representative gels from one of three biological replicates after cell growth at medium growth rates are shown. Strep- and His_6_-MBP-EV control purifications are presented in (**A**) and (**C**) and Strep-LpxC and His_6_-MBP-FtsH purifications in (**B**) and (**D**). The molecular weight of Strep-LpxC is approximately 34 kDa, of His_6_-MBP-FtsH about 114 kDa, and of His_6_-MBP about 44 kDa. Strep-LpxC, His_6_-MBP-FtsH and His_6_-MBP-EV control were successfully enriched and purified. The Strep-tag is too small for detection. Elution fractions E2 (Strep-LpxC) and E1 (His_6_-MBP-FtsH) contain the highest protein concentrations determined by the Bradford assay and were used for further experiments. Abbreviations: M = marker, S = supernatant, P = pellet, F = flow through, W = washing fraction, E = elution fraction, EV = empty vector, MBP = maltose-binding protein, Strep = streptavidin.


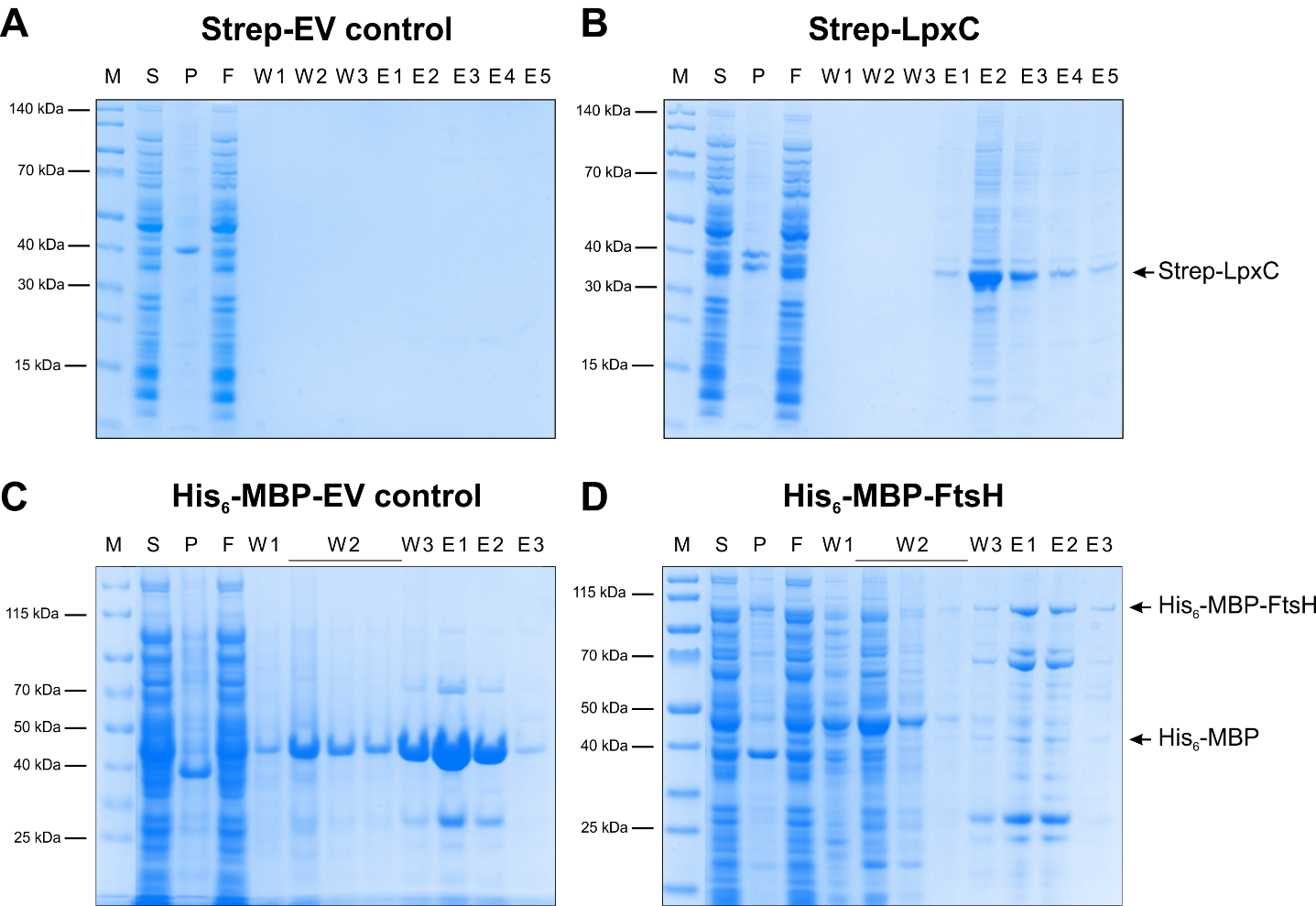

Supplement: Supplementary file 5 [file Data_Sheet_1.docx]
